# Development and validation of a nomogram to predict recurrence in epithelial ovarian cancer using complete blood count and lipid profiles

**DOI:** 10.3389/fonc.2025.1525867

**Published:** 2025-02-03

**Authors:** Xi Tang, Jingke He, Qin Huang, Yi Chen, Ke Chen, Jing Liu, Yingyu Tian, Hui Wang

**Affiliations:** ^1^ Department of Gynecology, The First Affiliated Hospital of Chongqing Medical University, Chongqing, China; ^2^ Department of Urology, The First Affiliated Hospital of Chongqing Medical University, Chongqing, China

**Keywords:** nomogram, epithelial ovarian cancer, recurrence, complete blood count, lipid profile

## Abstract

**Objective:**

Ovarian cancer is one of the most lethal gynecological malignancies. This study aimed to evaluate the prognostic significance of complete blood count (CBC) and lipid profile in patients with optimally debulked epithelial ovarian cancer (EOC) and develop a nomogram model to predict recurrence-free survival (RFS).

**Methods:**

This retrospective study analyzed patients diagnosed with EOC between January 2018 and June 2022.

**Results:**

A total of 307 patients were randomly divided into training and validation sets in a ratio of 7:3. Grade, International Federation of Gynecology and Obstetrics (FIGO) stage, platelet-to-lymphocyte ratio, red blood cell distribution width-coefficient of variation, triglycerides, and human epididymal protein 4 were identified as independent prognostic factors. The novel nomogram displayed a good predictive performance, with a concordance index (C-index) of 0.787 in the training group and 0.807 in the validation group. The areas under the curve for 1-, 3-, and 5-year RFS were 0.770, 0.881, and 0.904, respectively, in the training group, and 0.667, 0.906, and 0.886, respectively, in the validation group. The calibration curves exhibited good concordance between the predicted survival probabilities and actual observations. Time-dependent C-index curves, integrated discrimination improvement, net reclassification index, and decision curve analysis showed that the nomogram outperformed FIGO staging.

**Conclusion:**

This study established and validated a nomogram combining CBC and lipid profiles to predict RFS in patients with optimally debulked EOC, which is expected to aid gynecologists in individualized prognosis assessment and clinical management.

## 1 Introduction

Ovarian cancer (OC) is the third most common malignancy of the female reproductive system, following cervical and endometrial cancers. It is the fifth leading cause of cancer-related deaths and the primary cause of mortality from gynecological cancers among women (1). Epithelial ovarian cancer (EOC), the predominant pathological type, accounts for 85%-95% of all ovarian malignancies. Most patients with OC are asymptomatic in the early stages, with 70% presenting in advanced stages during their initial visit. Even after aggressive treatment and achieving complete clinical remission, 70% of patients relapse within two to three years following initial treatment, with a 5-year survival rate of only 25%-30% (2). Moreover, as patients experience recurrences and receive subsequent treatments, their recurrence-free survival (RFS) period often decreases with each recurrence (3). Therefore, prolonging RFS in patients with newly diagnosed OC is crucial. The inflammatory microenvironment of tumors promotes tumorigenesis and progression (4, 5). Assessing the inflammatory response is critical for cancer prognosis because it is reflected in changes in blood inflammatory markers (6). These markers can be measured using inexpensive and reliable complete blood count (CBC) tests and have been studied as prognostic factors in various cancers. Composite blood inflammatory markers such as the neutrophil-to-lymphocyte ratio (NLR), platelet-to-lymphocyte ratio (PLR), and monocyte-to-lymphocyte ratio (MLR) serve as prognostic factors for many cancers, including lung (7), cervical (8), and ovarian cancers (6). Recently, the red blood cell distribution width (RDW) has emerged as a potential prognostic factor for malignancies. However, its prognostic significance remains unclear and has been less studied in patients with OC.

Abnormal lipid levels may contribute to cancer progression through mechanisms such as obesity and systemic inflammation (9). Lipid profiles have been investigated as risk and prognostic factors in various cancers, including breast (10), prostate (11), and colorectal (12) cancers. However, the association between lipid levels and EOC has been less studied and remains unclear. Owing to the similarities between EOC, breast cancer, and prostate cancer (13), such as the involvement of the BRCA1 and BRCA2 genes, abnormal lipid levels may affect the risk and prognosis of EOC.

Nomograms are extensively used tools that integrate various prognostic and determinant variables to generate a numerical probability of clinical events in individuals, thereby advancing personalized medicine. Compared with traditional staging systems, nomograms provide rapid calculations through a user-friendly digital interface, offering greater accuracy and ease of interpretation. They enhance the assessment of individual risk, thereby aiding clinical decision-making and improving patient-physician communication (14). While previous studies have developed prognostic nomogram models for EOC, these models primarily rely on classical clinical parameters such as histological grade, pathological type, International Federation of Gynecology and Obstetrics (FIGO) stage, residual tumor size, and ascites. The studies mentioned above have shown that CBC and serum lipid levels are associated with the development and prognosis of malignancies. These parameters are easily accessible in the clinic, yet no studies have combined CBC and serum lipid levels to develop a prognostic nomogram model for EOC.

This study aimed to evaluate the combined impact of pre-treatment CBC and lipid profiles on the recurrence of EOC. By integrating these factors with classical clinical parameters, we sought to develop a nomogram model to predict RFS in patients with optimally debulked EOC.

## 2 Materials and methods

### 2.1 Study population

**Figure 1:**
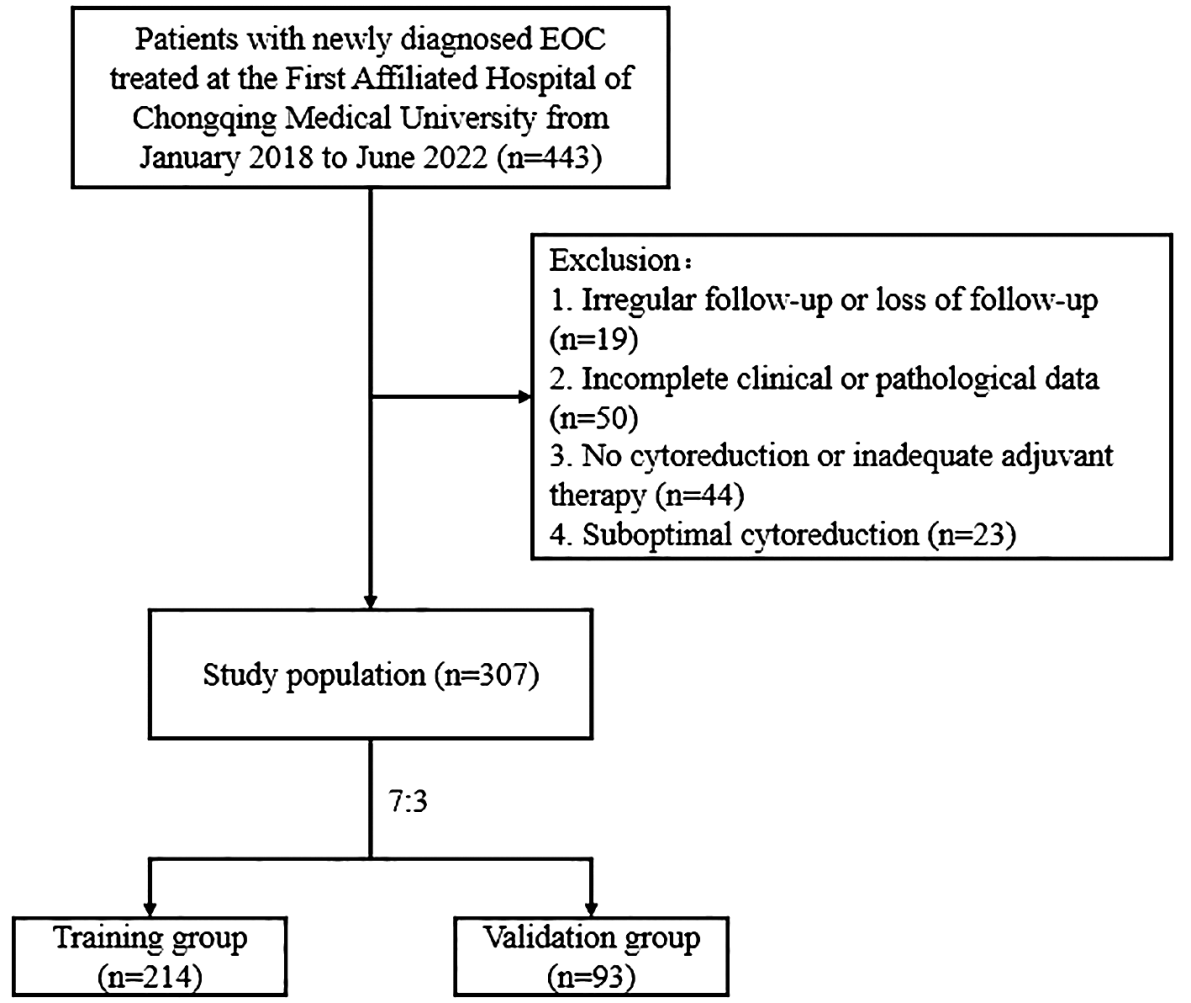
Flowchart of patients with optimally debulked EOC based on the inclusion and exclusion criteria.

Retrospective data were collected from patients newly diagnosed with EOC and treated at the First Affiliated Hospital of Chongqing Medical University between January 2018 and June 2022. Patients were followed up until recurrence or February 2024. The inclusion criteria were as follows: (1) histologically confirmed EOC; (2) optimal cytoreduction (residual tumor size <1 cm), followed by adequate adjuvant therapy, achieving complete clinical remission; and (3) availability of complete clinical and pathological data. The exclusion criteria were as follows: (1) incomplete clinical or pathological data; (2) no cytoreduction or inadequate adjuvant therapy; (3) suboptimal cytoreduction; (4) irregular follow-up or loss of follow-up; (5) RFS less than 1 month; (6) history of other malignancies; (7) presence of hematologic disorders, inflammatory diseases, or infections; and (8) death due to other diseases. This study was approved by the Ethics Committee of the First Affiliated Hospital of Chongqing Medical University and the need for informed consent was waived (2024-080-01). EOC recurrence was defined as serum cancer antigen 125 (CA125) levels exceeding the normal value (35 U/mL) or recurrent lesions detected on imaging. The study endpoint was RFS, which was defined as the time from the start of treatment to the earliest evaluation of the first recurrence. A flowchart of the study is shown in **Figure 1**.

### 2.2 Statistical analysis

R software (version 4.1.2) was used to perform all statistical analyses. The “survminer” package in R software was applied to determine the optimal cutoff values for each quantitative variable. Patient data were randomly divided into training and validation sets in a 7:3 ratio. Comparisons of categorical variables between groups were performed using the chi-square test or Fisher’s exact test. Comparisons between the groups of quantitative variables were conducted using the t-test for normally distributed data and the rank-sum test for non-normally distributed data. The nomogram was developed using a training set, and its evaluation and validation were performed using the validation set. In the training set, univariate Cox regression analysis was performed on all factors to identify prognostic factors associated with EOC recurrence. Factors with P < 0.05 were subsequently included in the multivariate Cox regression analysis to identify independent prognostic factors for EOC recurrence. Finally, a nomogram model was constructed based on these independent prognostic factors to predict EOC recurrence.

The discriminative ability and calibration of the nomogram were evaluated using the concordance index (C-index), receiver operating characteristic (ROC) curves, area under the curve (AUC), and calibration curves. Decision curve analysis (DCA) quantified the net benefit of the nomogram at different threshold probabilities and compared it with that of the FIGO staging system. The integrated discrimination improvement (IDI) and net reclassification index (NRI) were used to compare the predictive power and clinical benefits of the nomogram with FIGO staging. Based on the total score from the nomogram, a new recurrence risk stratification was established, categorizing patients into high- and low-risk groups. Survival differences between these risk groups were compared using the log-rank test and the Kaplan–Meier method.

## 3 Results

### 3.1 Patient clinicopathological characteristics

According to the inclusion and exclusion criteria, we enrolled 307 patients with EOC who were randomly assigned to the training (n=214) or validation (n=93) groups at a ratio of 7:3 (**Figure 1**). The median RFS was 30 months (8-71 months). Among these patients, 118 experienced recurrences, with 85 and 33 patients in the training and validation cohorts, respectively. The clinicopathological characteristics of the patients are summarized in **Table 1**. The clinicopathological characteristics were statistically similar between the training and validation cohorts indicating comparability between the groups.

**Table 1:** Characteristics of patients with optimally debulked EOC in training and validation groups.

	Total	Training group	Validation group	*P*
	N=307 (%)	N=214 (%)	N=93 (%)	
Age				1.000
≤41	32 (10.42%)	22 (10.30%)	10 (10.80%)	
>41	275 (89.58%)	192 (89.70%)	83 (89.20%)	
Recurrent				0.566
No	189 (61.56%)	129 (60.30%)	60 (64.50%)	
Yes	118 (38.44%)	85 (39.70%)	33 (35.50%)	
Grade				0.582
G1-G2	65 (21.17%)	43 (20.10%)	22 (23.70%)	
G3	242 (78.83%)	171 (79.90%)	71 (76.30%)	
FIGO				0.775
I	54 (17.59%)	38 (17.76%)	16 (17.20%)	
II	65 (21.17%)	46 (21.50%)	19 (20.40%)	
III	147 (47.88%)	99 (46.26%)	48 (51.60%)	
IV	41 (13.36%)	31 (14.48%)	10 (10.80%)	
Histological type				0.700
Serous	213 (69.38%)	144 (67.30%)	69 (74.19%)	
Endometrioid	24 (7.82%)	19 (8.88%)	5 (5.38%)	
Clear cell	35 (11.40%)	27 (12.60%)	8 (8.60%)	
Mucinous	24 (7.82%)	16 (7.48%)	8 (8.60%)	
Mixed	5 (1.63%)	3 (1.40%)	2 (2.15%)	
Other	6 (1.95%)	5 (2.34%)	1 (1.08%)	
Residual tumor size				1.000
R0	237 (77.20%)	165 (77.10%)	72 (77.40%)	
R1	70 (22.80%)	49 (22.90%)	21 (22.60%)	
Targeted therapy				0.211
No	246 (80.13%)	176 (82.20%)	70 (75.30%)	
Yes	61 (19.87%)	38 (17.80%)	23 (24.70%)	
PLR				0.438
≤210.68	183 (59.61%)	124 (57.90%)	59 (63.40%)	
>210.68	124 (40.39%)	90 (42.10%)	34 (36.60%)	
MLR				0.945
≤0.41	222 (72.31%)	154 (72.00%)	68 (73.10%)	
>0.41	85 (27.69%)	60 (28.00%)	25 (26.90%)	
NLR				0.431
≤1.973	72 (23.45%)	47 (22.00%)	25 (26.90%)	
>1.973	235 (76.55%)	167 (78.00%)	68 (73.10%)	
RDW.CV				0.209
≤12.3	64 (20.85%)	40 (18.70%)	24 (25.80%)	
>12.3	243 (79.15%)	174 (81.30%)	69 (74.20%)	
TC (mmol/L)				0.678
≤4.99	247 (80.46%)	174 (81.30%)	73 (78.50%)	
>4.99	60 (19.54%)	40 (18.70%)	20 (21.50%)	
TG (mmol/L)				0.872
≤1.56	228 (74.27%)	160 (74.80%)	68 (73.10%)	
>1.56	79 (25.73%)	54 (25.20%)	25 (26.90%)	
HDL (mmol/L)				0.241
≤1.21	169 (55.05%)	123 (57.50%)	46 (49.50%)	
>1.21	138 (44.95%)	91 (42.50%)	47 (50.50%)	
LDL (mmol/L)				0.843
≤2.06	59 (19.22%)	40 (18.70%)	19 (20.40%)	
>2.06	248 (80.78%)	174 (81.30%)	74 (79.60%)	
CA125 (u/mL)				1.000
≤327.10	130 (42.35%)	91 (42.50%)	39 (41.90%)	
>327.10	177 (57.65%)	123 (57.50%)	54 (58.10%)	
HE4 (pmol/L)				0.249
≤149.00	135 (43.97%)	89 (41.60%)	46 (49.50%)	
>149.00	172 (56.03%)	125 (58.40%)	47 (50.50%)	

R0, no residual disease; R1, residual disease<1cm.

### 3.2 Determination of cutoff values for quantitative variables

The “surv_cutpoint” function from the “survminer” package in R software (version 4.1.2) was used to determine the optimal cutoff values for quantitative variables. The identified cutoff values were as follows: age 41 years, PLR 210.68, MLR 0.41, NLR 1.973, red blood cell distribution width-coefficient of variation (RDW-CV) 12.3%, cholesterol (TC) 4.99 mmol/L, triglycerides (TG) 1.56 mmol/L, high-density lipoprotein (HDL) 1.21 mmol/L, low-density lipoprotein (LDL) 2.06 mmol/L, CA125 327.10 u/mL and human epididymis protein 4 (HE4) 149.00 pmol/L.

### 3.3 Univariate and multivariate analysis for predicting EOC recurrence

In the training cohort, univariate Cox regression analysis was performed and 12 variables were found to be associated with RFS in optimally debulked EOC patients, including age, histological grade (15), FIGO stage, residual tumor size, PLR, MLR, NLR, RDW-CV, TG, HDL, CA125, and HE4 (P < 0.05). These variables were subsequently analyzed using multivariate Cox regression, which identified six independent prognostic factors affecting RFS in this patient population (**Table 2**): histological grade, FIGO stage, PLR, RDW-CV, TG, and HE4 (P < 0.05). These six independent prognostic factors were integrated into a nomogram model to predict RFS in patients with optimally debulked EOC.

**Table 2:** Univariate and multivariate Cox analysis for predicting RFS in optimally debulked EOC patients.

Characteristics	Univariate analysis		Multivariate analysis	
	HR (95%CI)	*P*	HR (95%CI)	*P*
Age (year)				
≤41	Reference		Reference	
>41	3.67 (1.16-11.61)	**0.027**	1.16 (0.34-3.98)	0.812
Grade				
G1-G2	Reference		Reference	
G3	9.02 (2.85-28.57)	**<0.001**	3.71 (1.08-12.69)	**0.037**
FIGO				
I	Reference		Reference	
II	9.13 (1.17-71.31)	**0.035**	5.45 (0.68-43.65)	0.110
III	28.22 (3.90-204.25)	**0.001**	7.76 (0.98-61.64)	0.053
IV	44.53 (5.98-331.46)	**<0.001**	8.90 (1.06-74.40)	**0.044**
Residual tumor size				
R0	Reference		Reference	
R1	1.98 (1.26-3.11)	**0.003**	1.09 (0.67-1.76)	0.735
Targeted therapy				
No	Reference			
Yes	1.09 (0.63-1.88)	0.758		
PLR				
≤210.68	Reference		Reference	
>210.68	3.36 (2.15-5.26)	**<0.001**	1.79 (1.02-3.15)	**0.044**
MLR				
≤0.41	Reference		Reference	
>0.41	2.94 (1.91-4.53)	**<0.001**	1.10 (0.65-1.86)	0.717
NLR				
≤1.973	Reference		Reference	
>1.973	3.91 (1.80-8.48)	**0.001**	1.39 (0.58-3.32)	0.459
RDW.CV (%)				
≤12.3	Reference		Reference	
>12.3	0.58 (0.35-0.95)	**0.031**	0.54 (0.32-0.92)	**0.023**
TC (mmol/L)				
≤4.99	Reference			
>4.99	0.78 (0.43-1.41)	0.414		
TG (mmol/L)				
≤1.56	Reference		Reference	
>1.56	1.88 (1.20-2.94)	**0.006**	1.67 (1.02-2.75)	**0.042**
HDL (mmol/L)				
≤1.21	Reference		Reference	
>1.21	0.42 (0.26-0.68)	**<0.001**	0.72 (0.43-1.21)	0.211
LDL (mmol/L)				
≤2.06	Reference			
>2.06	0.87 (0.51-1.46)	0.588		
CA125 (u/mL)				
≤327.10	Reference		Reference	
>327.10	4.64 (2.65-8.12)	**<0.001**	0.90 (0.44-1.87)	0.787
HE4 (pmol/L)				
≤149.00	Reference		Reference	
>149.00	5.91 (3.20-10.91)	**<0.001**	2.44 (1.19-5.00)	**0.015**

R0, no residual disease; R1, residual disease<1cm.

P < 0.05 (in bold) was considered to be statistically significant.

### 3.4 Establishment and validation of the nomogram model

**Figure 2:**
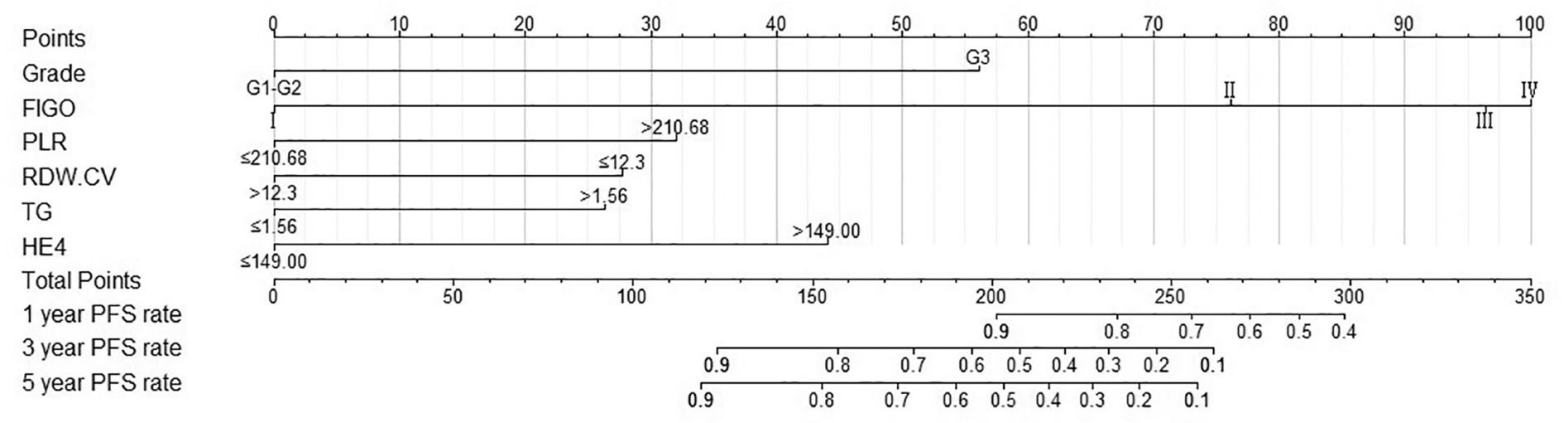
Nomogram to predict 1−, 3−, and 5−year RFS for patients with optimally debulked EOC.

**Figure 3:**
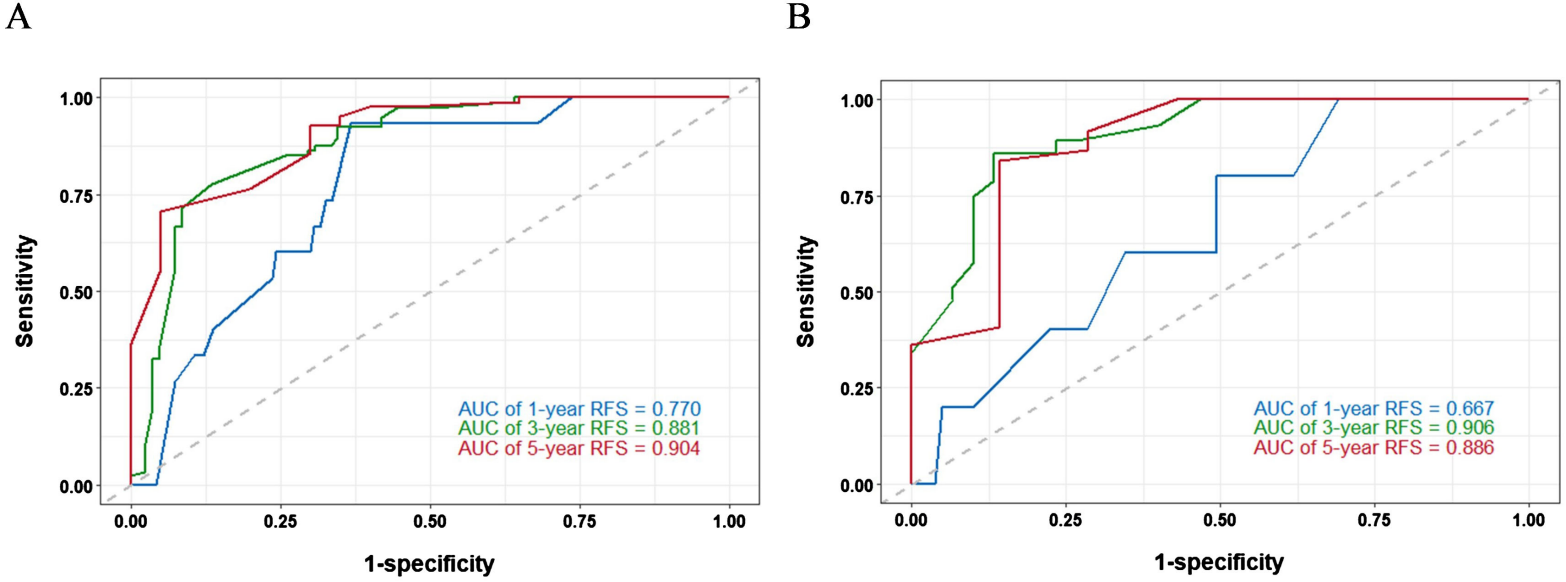
Time−dependent ROC curves of the nomogram for predicting 1−, 3−, and 5−year RFS in training set **(A)** and validation set **(B)**.

**Figure 4:**
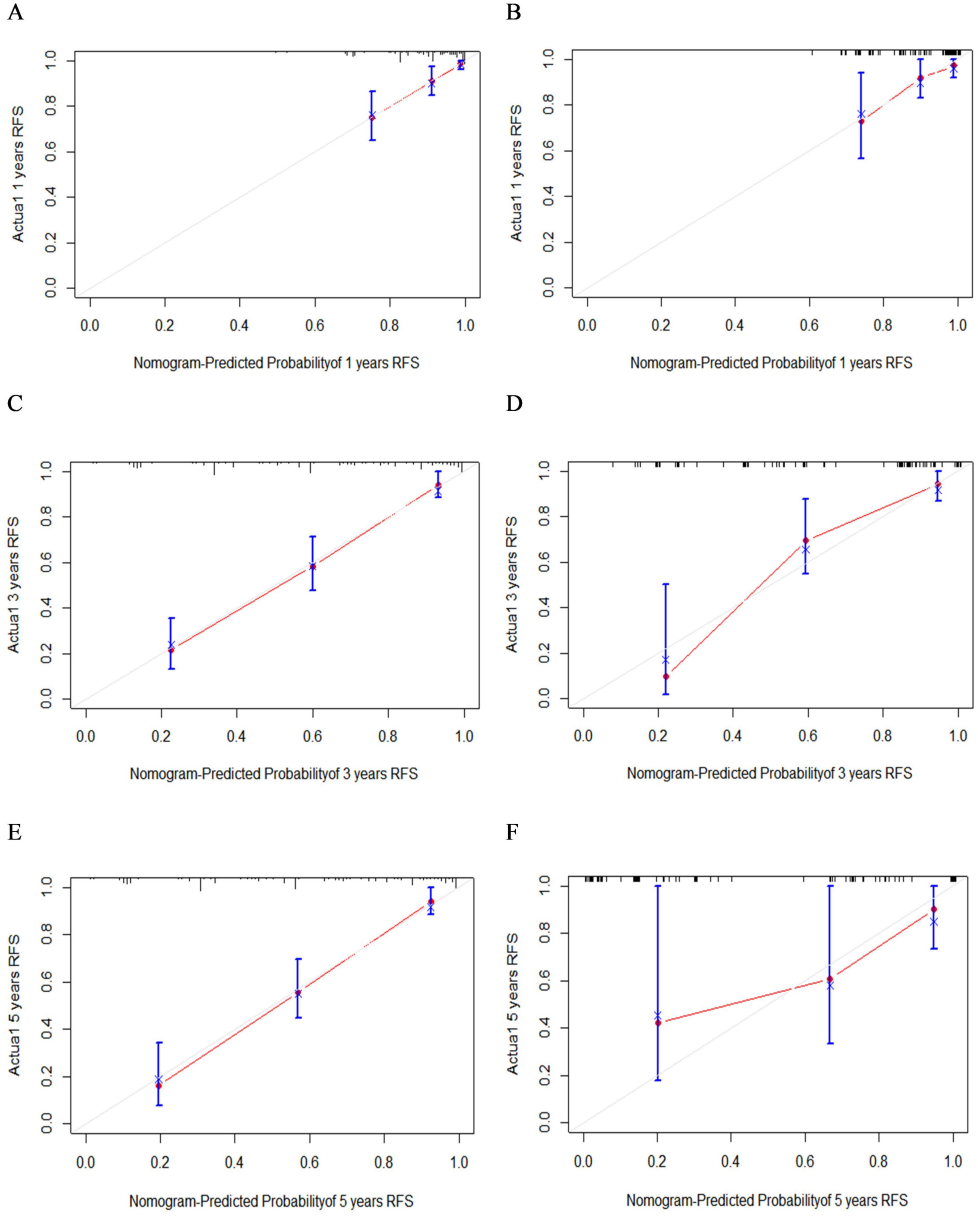
The calibration curves of 1- **(A)**, 3- **(C)**, and 5-year **(E)** RFS in the training group and 1- **(B)**, 3- **(D)**, and 5-year **(F)** RFS in the validation group.

**Figure 5:**
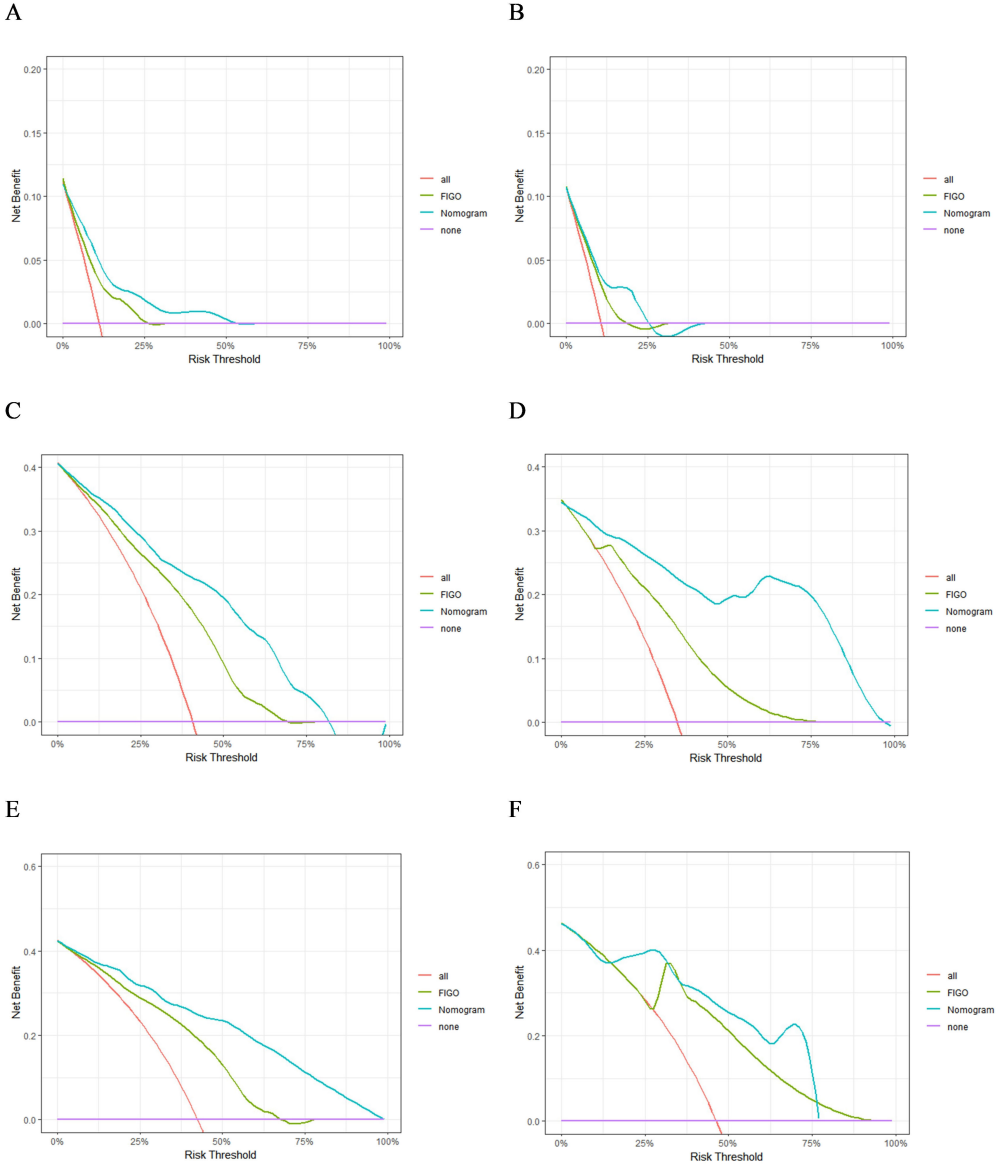
The DCA of 1- **(A)**, 3- **(C)**, and 5-year **(E)** RFS in the training group and 1- **(B)**, 3- **(D)**, and 5-year **(F)** RFS in the validation group.

A nomogram model was developed to predict 1-, 3-, and 5-year RFS in patients with optimally debulked EOC based on the identified independent prognostic factors (**Figure 2**). The total scores for all six variables were calculated to predict the individual 1-, 3-, and 5-year RFS rates. Additionally, a dynamic web-based calculator (Dynamic Nomogram) (https://beenle.shinyapps.io/dynnomapp/) was further prepared based on the nomogram using the “Dynnom” package. The discriminative ability and calibration of the nomogram were evaluated using the C-index, time-dependent ROC curves, and calibration curves. The C-index was 0.787 (95% confidence interval (CI): 0.743-0.831) in the training set and 0.807 (95% CI: 0.735-0.879) in the validation set (**Table 3**). Time-dependent ROC curves showed AUC values of 0.770, 0.881, and 0.904 for 1-, 3-, and 5-year RFS in the training set, and 0.667, 0.906, and 0.886 in the validation set, respectively (**Figure 3**), indicating the good discriminative ability of the nomogram. Moreover, the calibration curves demonstrated good consistency between the observed and predicted RFS in both sets (**Figure 4**). The DCA revealed that the nomogram model provided substantial net benefits over a broad spectrum of threshold probabilities (**Figure 5**).

**Table 3:** The NRI, IDI, and C−index of the nomogram and FIGO Stage system in RFS prediction for patients with optimally debulked EOC.

Index	Training group		*P*	Validation group		*P*
	Estimate	95%CI		Estimate	95%CI	
NRI (vs. FIGO)						
For 1-year RFS	0.131	-0.119-0.454	0.208	0.236	-0.127-0.620	0.184
For 3-year RFS	0.461	0.308-0.619	<0.001	0.411	0.111-0.744	0.014
For 5-year RFS	0.605	0.421-0.759	<0.001	0.317	0.019-0.898	<0.001
IDI (vs. FIGO)						
For 1-year RFS	0.021	-0.035-0.113	0.448	0.063	-0.004-0.279	0.072
For 3-year RFS	0.181	0.111-0.292	<0.001	0.198	0.079-0.368	0.006
For 5-year RFS	0.268	0.166-0.396	<0.001	0.162	0.015-0.454	<0.001
C-index						
The nomogram	0.787	0.743-0.831		0.807	0.735-0.879	
FIGO	0.706	0.657-0.755		0.706	0.635-0.777	

### 3.5 Comparison of the nomogram model and FIGO staging

**Figure 6:**
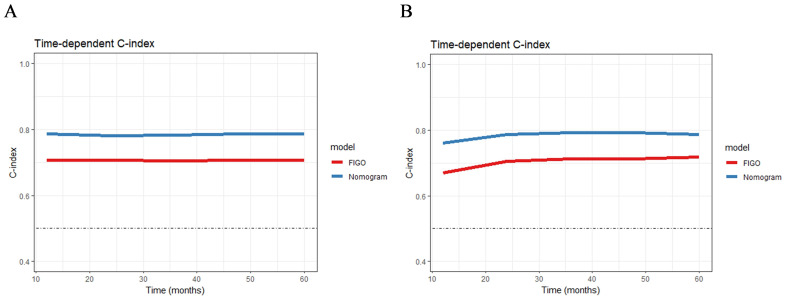
The time-dependent C-index curves of the nomogram for RFS prediction in training group **(A)** and validation group **(B)**.

The advantages of the new nomogram model over the traditional FIGO staging system were evaluated using C-index, DCA, NRI, and IDI. The time-dependent C-index curves indicated that the nomogram model exhibited superior discriminative ability compared with the FIGO staging system in both the training and validation groups (**Figure 6**). DCA analysis showed that the nomogram model provided greater clinical utility than the FIGO staging system (**Figure 5**). In NRI and IDI analyses, the nomogram model outperformed the FIGO staging system (**Table 3**). In the training set, the NRI values for the nomogram at 1-, 3-, and 5-year were 0.131 (95% CI: -0.119-0.454, P=0.208), 0.461 (95% CI: 0.308-0.619, P<0.001), and 0.605 (95% CI: 0.421-0.759, P<0.001), respectively, while the IDI values at these time points were 0.021 (95% CI: -0.035-0.113, P=0.448), 0.181 (95% CI: 0.111-0.292, P<0.001), and 0.268 (95% CI: 0.166-0.396, P<0.001). In the validation set, the NRI values at 1-, 3-, and 5-year were 0.236 (95% CI: -0.127-0.620, P=0.184), 0.411 (95% CI: 0.111-0.744, P=0.014), and 0.317 (95% CI: 0.019-0.898, P<0.001), respectively, while the IDI values were 0.063 (95% CI: -0.004-0.279, P=0.072), 0.198 (95% CI: 0.079-0.368, P=0.006), and 0.162 (95% CI: 0.015-0.454, P<0.001), respectively. Compared to the traditional FIGO staging system, our nomogram model showed a trend toward better predictive ability at one year, though the difference was not statistically significant. However, the nomogram demonstrated significant superiority in predictive ability at 3 and 5 years. These findings suggest that the new nomogram model demonstrates superior overall predictive accuracy compared with the traditional FIGO staging system.

### 3.6 Ability of nomogram to stratify patient recurrence risk

**Figure 7:**
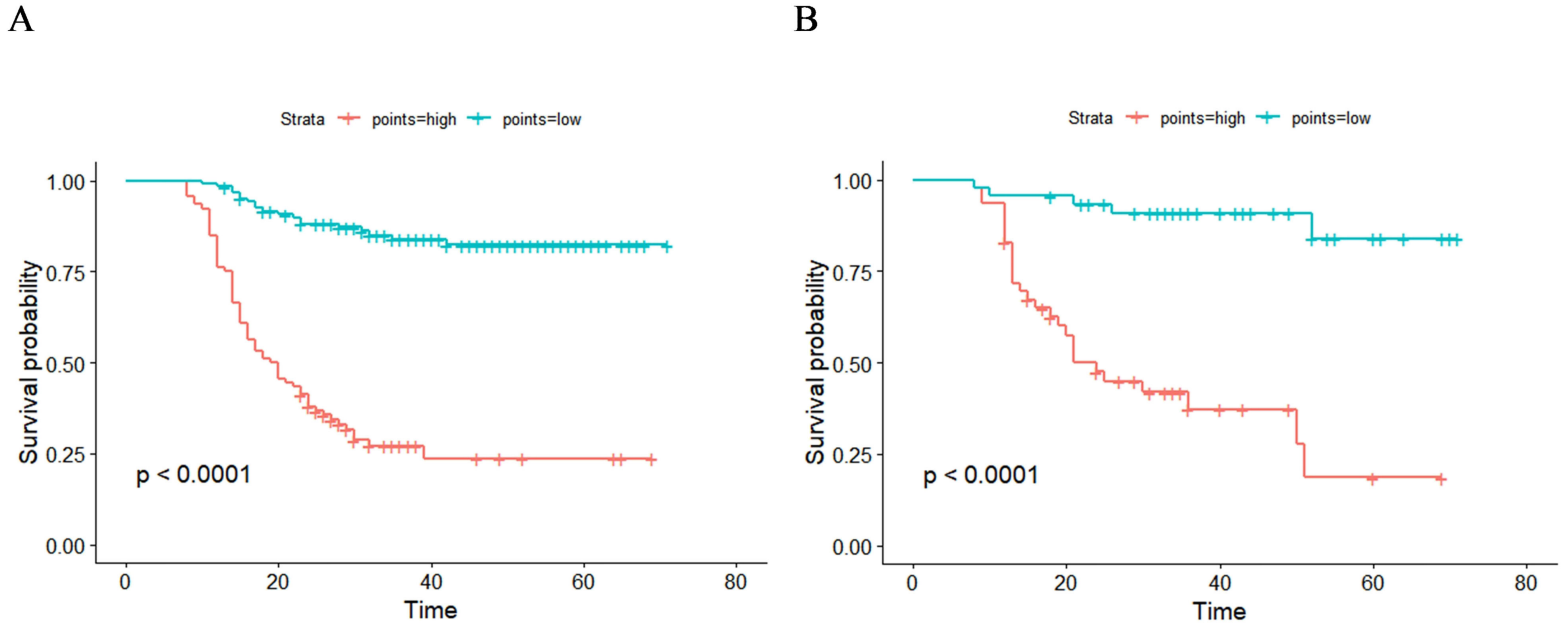
Kaplan–Meier curves of two recurrence risk subgroups in training group **(A)** and validation group **(B)** according to the new risk stratification system.

The total score for all patients was calculated based on the nomogram using the “nomogramFormula” package, and the optimal cutoff value for the total score was determined using the “surv_cutpoint” function of the “survminer” package. Patients in both the training and validation groups were then classified into high-risk (total score >196.58) and low-risk groups (total score ≤196.58). Subsequently, Kaplan–Meier curves and log-rank tests revealed a significant difference in RFS between the low-risk and high-risk groups across both the training and validation cohorts (**Figure 7**). Patients in the high-risk group were more likely to experience relapse than those in the low-risk group.

## 4 Discussion

In this study, we assessed the impact of pre-treatment CBC and lipid profiles on recurrence in patients with optimally debulked EOC. PLR, RDW-CV, and TG were found to be significantly associated with RFS. Other common prognostic factors identified included the FIGO stage, histological grade, and pre-treatment HE4 levels. Nomograms provide a highly accurate method for evaluating individual survival prognosis based on disease characteristics, facilitating clinical decision-making for patients with various types of tumors. Therefore, we developed and validated a nomogram incorporating these prognostic factors to predict the 1-, 3-, and 5-year RFS rates. Additionally, we created a dynamic web-based version of the nomogram which allows users to easily predict a patient's probability of recurrence and the 95% CI by inputting the patient's clinical characteristics. This tool can assist in clinical decision-making and enhance patient-physician communication. For high-risk patients with EOC, maintenance therapy with targeted agents, endocrine therapy, or immunotherapy after chemotherapy may help reduce the risk of recurrence and improve prognosis. Conversely, for low-risk patients, the frequency of follow-ups may be appropriately reduced without affecting patient prognosis, formulating individualized follow-up plans, and reducing follow-up costs.

Lipids are crucial metabolites that form a significant part of the cell membrane and participate in various cellular functions including energy storage, cell differentiation, and signal transduction. Abnormal lipid levels promote tumorigenesis, colonization, and metastasis. To metastasize, tumor cells must pass through various stages, involving metabolic and structural adaptations related to lipids, including changes in the lipid membrane composition to facilitate the invasion of new niches and evade mechanisms of cell death, along with increased lipid catabolism and anabolism for energy production and protection against oxidative stress. Cancer cells exploit lipid metabolism to regulate the activity of stromal and immune cells for their benefit, thus contributing to treatment resistance and promoting recurrence (16). Hypertriglyceridemia, a common form of dyslipidemia, is a recognized risk factor for cardiovascular disease. Several studies have found a significant correlation between elevated TG levels and adverse prognosis in non-small cell lung cancer (17). Similar findings have been reported in colorectal (13), breast (11), and cervical cancers (18). However, Li et al. found that low TG levels are significantly associated with poor disease-free survival in patients with breast cancer (19). Studies on TG levels in EOC are limited and controversial. Contrary to our findings, Li et al. reported that decreased TG levels are a specific metabolic feature that predicts early EOC recurrence (20). In contrast, Huang et al. found that high TG levels were associated with poor progression-free survival (PFS) in patients with EOC receiving bevacizumab targeted therapy (21). A case-control study of metabolism and OC in a Chinese population revealed that metabolic syndrome, including hypertriglyceridemia, was significantly correlated with poor PFS and overall survival (OS) in EOC (10). However, some studies have not found an association between TG levels and EOC prognosis (15). Our study found that pre-treatment TG levels above the cutoff value of 1.56 mmol/L were associated with worse RFS in patients with optimally debulked EOC. Differences in the study populations, follow-up periods, sample sizes, study endpoints, and statistical adjustments for confounding factors may account for these conflicting results. Fatty acids (FAs), one of the main components of triglycerides, are biosynthesized in the cytoplasm by fatty acid synthase (FASN). As a central regulator of lipid metabolism, FASN is essential for the proliferation and survival of lipid phenotype tumors, reconnecting tumor cells for greater metabolic flexibility to meet their high energy demands. FASN overexpression and hyperactivity are often associated with malignant growth and tumor advancement (22). However, the intricate mechanisms and the biological importance of these processes require further investigation.

Many cancers originate from sites of infection, chronic irritation, and inflammation (23), and the inflammatory microenvironment is a critical component of the tumor microenvironment (5), indicating a complex interplay between inflammation and cancer. Cancer cells stimulate platelet production and lead to platelet activation and aggregation, whereas platelets promote tumor growth, tissue invasion, and metastasis. Numerous platelet-derived factors are integral to the tumor microenvironment and contribute to cancer progression (24). Moreover, lymphocytes are vital for tumor defense, as they induce cytotoxic cell death and inhibit tumor cell proliferation and migration. PLR, a composite marker of hematologic inflammation, is a prognostic marker in various cancers (8, 9). An elevated PLR indicates the activation of transcription factors involved in the inflammatory response, such as nuclear factor-kB, signal transducer, activator of transcription 3, and hypoxia-inducible factor 1-alpha.These transcription factors synergistically produce some key pro-tumor growth cytokines, such as TNF-α, IL-1β, and IL-6 (25). Previous retrospective studies have confirmed the prognostic role of the PLR in EOC, which is consistent with our findings. For instance, Ceran et al. observed that the PLR was negatively correlated with OS in EOC (26). Plaja et al. reached the same conclusion (27). Additionally, a meta-analysis of 11 studies with 3574 patients indicated that a high pre-treatment PLR might be an adverse prognostic factor for clinical outcomes in patients with OC (28). Another meta-analysis of ten studies with 2919 patients also showed that a high PLR was associated with poorer PFS and OS (29). Our study found that in patients with optimally debulked EOC, a pre-treatment PLR above the threshold of 210.68 was associated with worse RFS.

The RDW reflects the heterogeneity of red blood cell volume and is traditionally used for the differential diagnosis of anemia. Recent studies have identified the diagnostic and prognostic value of RDW in cardiovascular diseases, venous thromboembolism, cancer, diabetes, community-acquired pneumonia, chronic obstructive pulmonary disease, and liver and kidney failure. Abnormal RDW may be associated with metabolic abnormalities including telomere shortening, oxidative stress, inflammation, poor nutritional status, dyslipidemia, hypertension, erythrocyte fragmentation, and altered erythropoietin function (30). Studies have identified RDW as a prognostic factor in various cancers; however, its prognostic role remains unclear due to conflicting results. Some studies have found that an elevated preoperative RDW is a significant predictor of poor cancer prognosis (31, 32), whereas other studies have shown that a high preoperative RDW is associated with better cancer outcomes (33, 34). Li et al. reported that a preoperative RDW of > 14.5% was a significant predictor of poor prognosis in EOC (35). Sastra et al. found that preoperative RDW > 13.19% was significantly associated with poor outcomes in patients with EOC (36), which is contrary to the findings of our study. However, some studies have indicated that high preoperative RDW is not an independent risk factor for poor prognosis in EOC (37). The discrepancies in these studies may be attributed to differences in study populations and sample sizes. Additionally, heterogeneity among different malignancies may influence the prognostic value of RDW. The clinical reference range for RDW is currently based on bilateral limits, and variations in the study population and statistical methods for determining the cutoff values could lead to different outcomes. In our study, the cutoff value for RDW-CV was 12.3%, and RDW-CV below this threshold was significantly associated with worse RFS.

In previous studies, the residual tumor size has been recognized as a significant prognostic factor for EOC. However, we observed that it was not associated with RFS in patients who underwent optimal debulking. This discrepancy may be attributed to adjuvant chemotherapy and targeted therapies administered postoperatively, which can potentially eliminate residual lesions < 1 cm in size, achieving effects comparable to those of complete surgical resection. Additionally, studies have shown that the size and distribution of postoperative residual lesions can influence both the timing and pattern of recurrence. Patients with complete surgical resection and residual disease <1 cm at a single anatomical site have similar rates of platinum-resistant recurrence, whereas those with residual disease <1 cm across multiple anatomical sites have an increased risk of platinum-resistant recurrence (38). Our study did not evaluate the distribution of the postoperative residual lesions in these patients. Previous studies have investigated the prognostic significance of microscopic residual disease following neoadjuvant chemotherapy (NACT) in patients with advanced EOC undergoing interval debulking surgery (IDS). These studies demonstrated that among patients who achieved R0 resection after IDS, those with more than three microscopic lesions post-NACT had a significantly shorter median OS and PFS than those with fewer than three lesions. Despite meticulous surgical efforts, eliminating microscopic residual disease hidden within chemotherapy-induced fibrotic areas is challenging (39). We did not explicitly differentiate between patients who underwent primary debulking surgery and those who underwent IDS. Therefore, among patients classified as having achieved R0 resection, some microscopic lesions may have been overlooked in the IDS.

To the best of our knowledge, this is the first study to evaluate the combined impact of pre-treatment CBC and serum lipid levels on EOC recurrence using a nomogram. Previous studies on EOC survival models have primarily focused on variables such as FIGO stage, CA125 levels, histological grade, pathological type, age, organ metastasis, and residual tumor size (40, 41). The significance of our study lies in the fact that CBC and lipid profile evaluations are low-cost and easily accessible clinical tests that can be repeatedly obtained through blood draws, allowing for dynamic monitoring. In addition to classic clinicopathological factors such as FIGO stage and histological grade, pre-treatment TG, RDW-CV, and PLR also serve as valuable prognostic indicators. Furthermore, apart from standard cancer treatments, patients with EOC may benefit from TG level adjustments. Patients with an abnormal PLR and RDW-CV may require more aggressive postoperative treatment and follow-up to reduce the risk of recurrence.

This study has several limitations. First, as this was a single-center retrospective analysis with a small sample size and no external validation, selection bias was inevitable. Additionally, our nomogram model did not include other relevant prognostic variables such as ascites, lymph node involvement, and organ metastasis, which limits the comprehensiveness of our research. Previously reported gene-related factors associated with OC prognosis such as homologous recombination repair deficiency (42) and KRAS/BRAF mutations (43) were excluded from the study. Furthermore, the preoperative comorbidities were not evaluated. To avoid bias and errors, future research should include external validation and larger and more diverse sample sources. Further prospective studies are needed to establish the optimal cutoff values for CBC and lipid levels.

## 5 Conclusion

This study identified histological grade, FIGO stage, PLR, RDW-CV, TG, and HE4 as independent prognostic factors for RFS in patients with EOC who underwent optimal cytoreduction. The nomogram model constructed based on these variables demonstrated good predictive performance and clinical value and was expected to predict recurrence and assist gynecologists in providing individualized prognostic assessments and clinical management for patients. However, further validation and refinement of the model are required to enhance its clinical applicability.

## Data Availability

The raw data supporting the conclusions of this article will be made available by the authors, without undue reservation. Researchers interested in accessing these data for non-commercial research purposes are encouraged to contact the corresponding author.
